# The ghost in the data: Evidence gaps and the problem of fake drugs in global health research

**DOI:** 10.1080/17441692.2020.1744678

**Published:** 2020-03-31

**Authors:** Sarah Hodges, Emma Garnett

**Affiliations:** a Department of History, University of Warwick, Coventry, UK; b School of Population Health and Environmental Sciences, King’s College London, London, UK

**Keywords:** Fake, pharmaceuticals, evidence, global health publishing, culture of scientific knowledge

## Abstract

For the past several decades, global health research and policy have raised the alarm about the growing threat of counterfeit and low-quality drugs (henceforth ‘fakes’). These high-profile and regularly-repeated claims about ‘fake drugs’ pepper scholarly publications, grey literature, and popular writing. We reviewed much of this work and found that it shares two characteristics that sit awkwardly alongside one another. First, it asserts that fake drugs constitute an urgent threat to lives. Second, it reports trouble with ‘gaps’ in the evidence on which their claims are based; that data is weaker and less conclusive than anticipated. Given the ubiquity of and urgency with these claims are made, we found this juxtaposition perplexing. To understand this juxtaposition better, we undertook a close reading of the strategies authors employed to negotiate and overcome data and evidence ‘gaps’ and asked questions about the cultures of scholarly publishing in global health research. We argue that a scholarly commitment to studying fakes despite--rather than because of—the evidence functions to support the continuation of similar research. It also works against asking different questions—for instance regarding the lack of easy access to pharmacological data that might make it possible to know fakes differently.

## Introduction

In March 2012, *The Lancet* ran the editorial ‘Counterfeit drugs: A growing global threat’ (Lancet 2012). The piece recounted recent seizures of bad drugs in the US, and went on to warn that, although concerns about drug safety previously focused on Africa and Asia, the globe's entire drug supply chain was now under threat. The concerns raised in the article are not new. Over the last few decades, research and policy have raised the alarm about the danger that fake pharmaceuticals pose to global health.

This narrative – that fake drugs threaten health – has been repeated so often and with such certainty that it has come to seem common-sensical. To explore the evidentiary basis of these concerns, we subjected these high-profile and oft-repeated claims in this body of published work to close scrutiny, following the paper trail of citation: sources, data, and experts. The upshot: Our reading of the published evidence points to a more complicated picture.

This scholarship maintains that fake drugs constitute an urgent problem for global health because fake drugs constitute an immediate threat to lives (e.g. Glass, 2014; Harris et al., 2009; Khan & Khar, 2015; Mackey et al., 2011, 2015). Such assertions are often treated as self-evident. For example, one frequently repeated claim is that India produces a staggering 75% of the world's counterfeit drugs, yet this piece of information is not attributed to any source (e.g. Khan & Khar, 2015, p. 2; Verma et al., 2014, p. 141). At the same time, the same papers report surprise at how little convincing evidence they found to support their claims about the urgent dangers presented by fake drugs (see also Rahman et al., 2018). This situation is not unique to the problem of fakes and we suspect that a similar reading of the published literature of other public health issues would result in similar findings. Our purpose is not to deconstruct how claims are made but rather investigate the ways in which fake drugs have become a widely accepted concern in global public health research. In particular, we are responding to calls to inform the development of new methodologies and approaches to fakes and suggest that better understanding cultures of knowledge production are key to this learning process.

This paper has three parts. First, we report on our initial findings from reviewing the literature. We recount the substantial mismatch between (a) the strength of claims about the prevalence and dangers of fake drugs, and (b) the recognition that fakes are difficult to measure, evidence and conceptualise. Second, we consider the rhetorical strategies that papers employed to negotiate and overcome problems with the evidence in order to make claims about the extent of the problem of fakes. Third, we explore the implications of this mismatch for the politics of knowledge, asking: What might these ‘evidence gaps’ tell us about the cultures of knowledge in the production, circulation, and consumption of scholarly literature itself? In particular, we note that responses to the challenges of generating data and evidence of fakes has been two-fold. We highlight a series of ‘workarounds’ used by scholars in their research and the reporting of fakes – that is, methods for circumventing problems with available data and evidence. From there, we show how these workarounds emerge in lieu of asking corollary questions about access to data on pharmaceuticals’ quality, such as those held by states or manufacturers.

Our approach focuses attention on the politics of knowledge. That is, rather than taking a problem – such as fake drugs – as self-evident, we join others from history, sociology, anthropology, and science and technology studies who investigate the conditions that allow a phenomenon to become an object of concern (e.g. Latour, 1987; Reinarman, 1988; Rekdal, 2014). In so doing, we ask: what can we discern about the cultures of knowledge at play in the scholarship on fake drugs? We argue that the upshot of the persistent mismatch between bold claims and weak evidence is to frame fake drugs as an *unknowable* problem. Rendering fake drugs as unknowable matters, we argue, because it misdirects (Peeters Grietens et al., 2019) attention away from the conditions that produce drugs’ unknowability – that is, the structural conditions that shape what we know and the ‘closed shop’ that is the pharmaceutical industry's approach to their own data (Sismondo, 2018).

Global public health scholarship on pharmaceuticals has attended to the problem of fake drugs for the past few decades. Alongside this, intellectual property legal thinkers, such as those associated with Third World Network, have produced large numbers of pamphlets, books, memos and online resources as part of campaigns that criticised anti-counterfeit policing of pharmaceuticals as this policing emerged as part of international trade regulations at the close of the twentieth and into the twenty-first centuries (https://www.twn.my/publications_ipr.htm). Nevertheless, this substantial attention to fake drugs has focused our collective critical gaze almost exclusively onto the operations of formal regulatory regimes. Our approach departs from these concerns.

Instead of exploring the efficacy or politics of formal regulatory regimes, in this article we take inspiration from recent scholarship in critical global health. This new body of work asks if it is in fact possible to distinguish clearly between the ‘real’ and the ‘fake’ even though these categories structure so much of the design, delivery, and experience of global health (Kingori & Gerrets, 2019; see also all of the accompanying articles in their guest-edited special issue). We also locate our intervention within the lively scholarly subfield of the anthropology and history of pharmaceuticals, where scholarship are asking vital questions about the production, circulation, and consumption of pharmaceuticals (Dumit, 2012; Greene, 2014; Hardon & Sanabria, 2017; Hayden, 2007, 2010, 2013; Kamat & Nichter, 1997, 1998; Nichter & Vuckovic, 1994; Peterson, 2014; Petryna et al., 2006; Sarojini et al., 2010; Sunder Rajan, 2017; van der Geest, 1982; van der Geest et al., 1996). This scholarship tracks the social lives of pharmaceuticals. It tells stories of their potential and of broken promises. It powerfully illuminates the uneven global distribution of pharmaceuticals’ therapeutic benefits.

Within broader medical humanities enquiry, questions of what is at stake in claims of pharmaceuticals’ fakeness, however, have only just begun to attract substantial attention (Cloatre, 2016; Gryseels et al., 2019; Hodges, 2019; Hornberger, 2018, 2019; Meek, 2018; Quet, 2018). Julia Hornberger's ethnographic analyses of the material and discursive careers of fake drugs is particularly important (2018, 2019). Hornberger explores how everyday practices of anti-counterfeit policing are rooted in the belief that large criminal conspiracies are to blame for the circulation of bad pharmaceuticals. She shows how this belief has, in turn, fuelled extensive funding for anti-counterfeit policing and products, such as the holograms or the barcodes that are now included as part of pharmaceutical products’ packaging. In particular, Hornberger's work shows how as the markets for – and the profitability of – anti-counterfeit policing products have grown, their chief observable effect has not been to make the global drugs supply chain safer, but instead to promote ever more wide-spread suspicions about the safety of *all* pharmaceuticals. By exploring the distribution of suspicion, Hornberger has opened up scholarly inquiries of fakeness as part and parcel of pharmaceuticals’ social lives.^1^

## Methodology: Conceptual tools and practical tactics

We write from our positions in the humanities and qualitative social sciences. We are invested not only in the epidemiological outcomes of global health practices, but also in the epistemological politics of global health knowledge. In order to explore the politics of knowledge, in this paper we understand global public health research on fake drugs as not simply reporting knowledge but also constituting it (Cohn, 1996; Hull, 2012; Sismondo, 2018). Our paper seeks to answer the questions: What is the underlying logic that makes these scholarly claims make sense? How do they, in fact, perform a particular reality? To ask questions of the culture of knowledge allows us to take a step back from the purported self-evident nature of the problem of fake drugs.

We focus in particular on the cultures of knowledge that shape how evidence is generated and framed in order to make claims about fakes. This involved analysing the discursive mechanisms by which uneven or ambiguous data and evidence about fake drugs is converted into global health claims. We trace how the material discursive work of scholarly practice constitute wider rhetorical patterns and are part of a rich discursive ecology that structures research of the contemporary global circulation of pharmaceuticals. To do this, our approach draws on two sets of scholarly traditions, and performs methods that in many ways mimic much of the research we analyse: the ‘desk review’. Based on a review of published findings we apply the methodological tools of ‘close reading’ and ‘reading against the grain’ that characterise much research on what has come to be seen as the ‘politics of knowledge’ across our respective disciplines: sociology and anthropology (author 2) and cultural history (author 1) (e.g. Bell, 2015; Cohn, 1996; Mkhwanazi, 2016). This methodology is particularly good at surfacing both the explicit and implicit assumptions that frame scholarly practice and its outputs. Focusing on rhetorical strategies of argumentation in scientific papers (Latour, 1987) is an opportunity to illuminate the broader culture of knowledge that this body of scholarship both draws on and reproduces.

The paper takes inspiration from those who have begun to explore the scholarly preoccupation with fakeness to discern the story it tells us about the production, circulation, and consumption of scholarly research itself. Whereas Dumit (2012) uses a methodology of close reading of claims about pharmaceutical advertisements, in this article, we identify patterns of argumentation within scholarly writing about fake pharma and read these patterns ‘against the grain’. In short, by employing this methodology we bring one set of conceptual framings to bear upon a body of scholarly research that explicitly pursues different ends.

We sought to take a snapshot of the literature, which means our sample is necessarily indicative rather than comprehensive (see Appendix for a full list of sources consulted in the review). Unlike the ‘systematic review’ that seeks to represent the literature that has been published, our aim is to demonstrate how the careers of certain problems have been sustained. The analysis we present also anticipates the modes by which cultures of scholarly publishing will continue to participate in its construction, and what as a result it might foreclose in terms of understanding and interrogating the social and political conditions of fakes.

By performing the methods of the studies we reviewed and analysed, we could understand how the problem of fakes is constructed and circulated through data practices, for instance, by sampling techniques, through tracing relationships between data sets, and by paying attention to citations and references in publications. Indeed, because many of the articles we studied explicitly discussed their search terms and search engines, we ran the same search terms (e.g. ‘fake’, ‘counterfeit’, ‘substandard’, ‘spurious’, ‘falsified’, and so on) through the same search engines (e.g. PubMed, Google Scholar, etc.) to create our sample: 42 scholarly articles all identified over several months in late 2017 and early 2018. Additionally, we augmented our initial results by soliciting suggestions from our informal scholarly networks. Finally, we ‘followed the paper trail’; we took an initial sample and read all of the pieces carefully for the terms contained in their references and bibliographies. We then added these to our sample as well (see Appendix for a full list of all literature collected and consulted).

Analysis involved identifying patterns in the rhetorical and material strategies by which ‘evidence gaps’ were worked around in the selected articles for review. This was followed by a close reading of examples of rhetorical strategies together and comparatively, in order to examine the politics of knowledge at play in these. We then considered what narratives about fakes were performed through the interpretive work of negotiating and overcoming ‘evidence gaps’: What understandings of fakes did they open up? Which did they foreclose?

## Findings: How the published literature negotiate and manage evidence gaps

Broadly, we found that all publications claimed fake drugs were a prevalent and growing problem for global health. Such claims were often followed by corollary assertions about the urgent dangers to health fakes pose. However, when we subjected the claims made in scientific publications to closer scrutiny we found persistent ‘evidence gaps’, which were also acknowledged in the published literature. Our identification of these ‘gaps’ matter because, in our analysis they function as empirical examples of the ways in which research and findings on fakes are produced, circulate and gain traction. Philosopher, sociologist and anthropologist Bruno Latour argues that a key rhetorical strategy in science is ‘bringing others in’ through citation practices that can help build an ‘argument from authority’ (1987, p. 31). In what follows, the discursive strategies of scholarly publishing are not considered solely in terms of how accurately they represent ‘the problem of fake drugs’ but in terms of the relations that materialise and mobilise the problem of fakes as a global health concern. For instance, the literature has many potential effects in terms of future research, the distribution of resources and funds, and regulations and legislation, among others. Although our focus is primarily on peer-reviewed scholarship, we found similar rhetorical strategies in grey literature. In what follows we describe four rhetorical patterns to overcome and make sense of ‘evidence gaps’ in research, and point to the implications of these practices and strategies on how the problem of fakes has become a concern and priority for global health.

### Reporting ‘evidence gaps’

The following is typical in an opening or conclusion of a scientific article on fakes, wherein the paper emphasises the absence of data on fake drugs: ‘Unfortunately, reliable information on the true public health and socioeconomic impacts of substandard and falsified medical products is sparse’ (WHO, 2017a, p. 1). In an even more detailed way, one report details how the extent of ‘data gaps’ means the generation of credible statements about fakes subsequently limited:

The original intention was to develop a simple model to allow for ‘guesstimated’ quantification of the extent of the contribution made to the development of antimicrobial resistance by poor quality medicines. However, the data gaps are too extensive to allow for any credible estimates to be developed. (Pisani, 2015, p. 4)

This declaration struck us as particularly significant when it was made in articles that were reviews of published scholarship (like our own). For example, there are published articles on the scale and nature of the problem of fake drugs through conducting search-based literature reviews using PubMed (Koczwara & Dressman, 2017; Rahman et al., 2018). In these specific cases, it was concluded that it was not possible to make a reliable statement about the prevalence of counterfeit drugs. Koczwara and Dressman point to the ‘heterogeneity of the results’ (2017, p. 2921) – a euphemism for what we refer to as ‘evidence gaps’. In another paper, it is stated that: ‘many of the reports identified in our study only provided seriously inadequate or even conflicting data’ (Rahman et al., 2018, p. 1300).

Evidence gaps were identified in scientific publications, but they were also materialised by reporting on conditions that might ‘host’ the missing evidence. Whilst signalling the urgency of the problem of fakes, in terms of the threat fakes pose to life-threatening conditions that can only be treated with life-saving medicines, for instance, many papers drew on documented cases of ill health caused by bad drugs that were not life-saving drugs. Instead they were products such as skin lighteners (see Hornberger, 2018 for a discussion of this). In other papers, fakes of both medical and non-medical products were presented and their problem defined in terms of their availability on the market. For example, one paper paints a picture of how fakes – both medicines and non-medical products – were presented to inquisitive passers-by and presumably potential consumers:

Such exposure presents a major health challenge as the market is likely to be proliferated with SF [substandard & falsified] pharmaceutical products for major diseases such as antiretroviral therapy (ART) anti-tuberculosis (anti-TB), anti-malarial therapy and other essential medicines. (Gwatidzo et al., 2017, p. 82)

Here, the possibilities of the market extend claims about fakes’ threat to health by using the extent of their reach (Hornberger, 2018) as indicative of ‘evidence gaps’. Similar claims were made about the absence of regulation in online markets (Interpol, 2014; see also discussion by Clark, 2015). Trouble controlling the global drug market has been used as a signal for the possible extent and availability of fakes to innocent consumers.

### Supplementing evidence

Although we were surprised at the ubiquitous acknowledgment of ‘evidence gaps’ in scientific publications, we found these statements did not always lead to a corollary questioning or moderation of claims about the prevalence of fake drugs. Instead, we observed a series of material and discursive ways of accounting for the ambiguity, uncertainty, and inaccessibility of data. Four ways in which the published literature used data to supplement the heterogeneous evidence base were identified: (a) refashioning multiple data; (b) sourcing data beyond biomedical research; (c) circumventing absent data with small samples and the ‘extreme case;’ and by (d) connecting fakes to other contemporary global health challenges, specifically anti-microbial resistance, for which there is data and evidence to build on.

#### Refashioning multiple data

Our analysis highlighted how the absence of data and evidence for a causal relation between fakes and health led to the pooling of different forms of data, gathered from a variety of sources and through a range of methods (e.g. Almuzaini et al., 2013; Khan & Khar, 2015; Koczwara & Dressman, 2017; Medina et al., 2016; Wertheimer & Norris, 2009). Bringing together diverse forms of data to make knowledge claims is not necessarily problematic, however the way in which they were put into dialogue created some comparisons that were insightful for understanding the cultures of knowledge production. For instance, in some publications data practices included combining crime data, field site sampling, mortality statistics, and results from lab studies to justify the extent of the problem of fakes (Kaur et al., 2016; Mackey & Liang, 2011). Individually these distinct forms of evidence were often shown to be limited in some way. Another example is a paper that detailed the development of screening devices for testing the molecular composition of drug samples to create a gold-standard for testing fakes (Kaur et al., 2016). This suggests that at the time of the research there were no effective tools of measurement. Other articles indicated that characteristics of a product, like packaging, or the conditions of places where medicines are sold, qualified something as fake, in lieu of evidence of the absence of an active pharmaceutical ingredient (Bate et al., 2013; Fatokun, 2016; Shukla & Sangal, 2009). The process of pooling and collating different kinds of data – refashioning data – was therefore a key part of building claims about fakes, through new material discursive compositions of the problem.

#### Sourcing data beyond scientific and biomedical literature

Secondly, demonstrating the presence of fakes involved the inclusion of undocumented sources, particularly with media reports and various journalistic accounts of fakes (e.g. Rahman et al., 2018; Stevens & Haja Mydin, 2013). A survey of published literature notes that: ‘a significant part of the published evidence regarding counterfeit antimicrobials derived from journalism rather than the biomedical literature’ (Kelesidis & Falagas, 2015, p. 459). Some publications relied very heavily on journalism to document fake drugs, for example, the study by the Interpol Pharmaceutical Crime Directorate (2014). Following scholarly articles’ reference lists, we went on to read the journalism cited but could not always find the data sources listed. This is not surprising or highly specific to this field, but is rather indicative of a mechanism by which narratives about fakes continue to grow and reproduce through academia (Rekdal, 2014).

#### Circumventing absent data: ‘Small’ data and the extreme case

The way in which fakes are reported in journalism, such as by developing narratives through examples of specific instances of the fake drug industry's existence or the harmful effects of fakes, was in some ways reflected in how they were reported in scientific publications. We noted the frequent use of what we call the ‘extreme case’ in our analysis. The extreme case is a version of small – or inapposite or under-documented – data samples serving as evidence for claims about a larger problem. For example, in one article it was suggested that rather than extrapolation, presumably through statistical methods, small and ‘unrepresentative’ data can offer insight into the problem of fakes:

… data analysis and samples collected by investigators in some of these studies were not necessarily representative of a large target area, and thus the prevalence obtained cannot be extrapolated to the whole country studied. However, these studies give an insight into the problem and, following our assessment of methodology, give the best evidence currently available in the literature. (Almuzaini et al., 2013, p. 6)

Small cases feed into a methodology of recirculating published literature that at once accepts the absence of evidence while claiming the existence of the problem of fakes. Small cases become key points in building a narrative understanding about the global circulation of fakes. For instance, when fake cancer drugs are discovered, or reports of local outbreaks of disease due to available drugs with no active pharmaceutical ingredient are found (Nayyar et al., 2015). ‘Small’ data is not necessarily a problem, and indeed qualitative social science research often draws on situated case studies to build empirical and theoretical claims. Yet rather than reflect on the problem-definition or methodology of research into fakes, the small-case was often framed as evidence in the absence of better evidence. Indeed, one major briefing report referred to a survey of medicines on sale at a large bazaar in New Delhi which found that only 7.5% were genuine but where this percentage came from was unclear (Stevens & Haja Mydin, 2013). The same report then cites a news article stating that twenty-two Indian pharmaceutical companies have been backlisted from exporting, importing and distributing drugs in Nigeria (Stevens & Haja Mydin, 2013, p. 5). Here, drugs from India are linked to pandemics in Africa through data from highly situated *and* diverse data, and despite an explicit discussion of the absence of precise data.

#### Connecting fakes to drug resistance

Another way in which ‘evidence gaps’ were worked around was by framing fake drugs as a possible driver or intensifier of drug resistance. In our review, we found the logic that connects the relationship between fakes and the existence of anti-microbial resistance to be deductive. That is to say, the claim that fake drugs are readily available is used as a starting point for understanding the existence of drug resistance (e.g. Bate et al., 2013; Newton et al., 2017).

The link between fake drugs and drug resistance was made by arguing that repetition of correlation evidence of causal links: ‘Scientific theory and common sense thus both suggest an inevitable link with [fake drugs and] antimicrobial resistance’ (Pisani, 2015, p. 12). Other articles discounted evidence of efficacy when active pharmaceutical ingredient (API) doses were lower than expected, claiming that the issue was not actually efficacy but long-term effects of drug resistance: ‘While these drugs may work in some cases, the insufficient API dose may prevent patients’ chances of cure and contribute to drug resistance’ (Bate et al., 2013, p. 3). With greater attention being paid to the question of drug resistance (particularly in the case of life-saving drugs), our research suggests that drug resistance enables the possibility of an *effect* of fake drugs to be evidenced empirically. For instance, emerging research suggests that poor quality medicines, like fakes, might be contributing to the development of drug-resistant pathogens in lower-income countries (Pisani, 2015, p. 39; see also Pisani, 2016).

Similarly, presenting evidence of resistance in sites where there is a lack of regulatory infrastructure was another way to demonstrate the problem of fake drugs (e.g. Banerjee, 2015). As one group writes: ‘[a]lthough a causal relation between poor quality artemisinin derivatives and artemisinin resistance has not been confirmed, modelling analyses suggest that under-dosing patients can play an important part in the spread of resistance’ (Nayyar et al., 2012, p. 488). By materialising data about fakes through modelling, a narrative about fakes, low doses of antibiotics, and possible resistance is subsequently mobilised. Introducing the concept of drug resistance was a way to infer underlying evidence exists. Speculative and theoretical work that sought to evidence a relationship between fakes and AMR is a necessary part of scientific inquiry. We are not criticising this approach but rather highlighting that what the ‘gap’ is in evidence is clearly unstable. Yet it is important to note that one of the effects of this new line of inquiry is to deflect attention away from this unstable evidence base.

### Uncertain data as implicit evidence of hidden data

By detailing these four discursive strategies that sought to compensate for ‘evidence gaps’ we have shown how they were used to demonstrate the existence of problem. Instead of treating ‘evidence gaps’ as evidence of a *lack* of evidence, gaps *themselves* also became evidence of the need to generate more evidence. This is particularly the case when concern that attention to the dangers of fakes may diminish as a result of the absence of evidence was expressed. For instance, having explained that data gaps were too extensive to allow for modelling, one paper argues that whilst ‘overall the picture is one of great uncertainty … this is overwhelmingly more likely to be because of a lack of representative information than the lack of a significant problem’ (Pisani, 2015, p. 3). We characterise this assertion as ‘uncertain data as implicit evidence of hidden data’ and point to the structural effects that uncertainty plays in determining how scientific resources are distributed in order to fill ‘knowledge gaps’. As Kelly et al. (2020) highlight, clinical and public health uncertainty define global health problems in ways that reflect wider cultures and power dynamics.

In short, we suggest that despite uncertain data and evidence there is a collective refusal of the possibility that fakes themselves might not exist, or at least not exist in the ways they are currently imagined. Further, ‘data gaps’ became evidence of the purposeful hiding of data. For example, some authors suggested that weak or uncertain data was an effect of criminal elements outsmarting surveillance mechanisms: ‘Despite clear global public health threats, surveillance for counterfeit medicines remains extremely limited, with available data pointing to an increasing global criminal trade that has yet to be addressed appropriately’ (Mackey & Liang, 2013, p. 233; also see Nayyar et al., 2015). Similarly, in a different paper it is stated that: ‘Only few published data admit the extent of the problem and its influence on public health. Thus, there is requirement of immediate attention and research by the regulatory authority towards this public safety issue’ (Khan et al., 2015, p. 5). We found that the published literature often asked open-ended questions, such as ‘How big is the problem?’ as a way to *indicate* the scale of evidence their research has not yet been able to document (Stevens & Haja Mydin, 2013, p. 2). In contrast, however, we found no one asking, ‘How *small* is the problem?’

### The trouble with categories

Finally, another way that the scientific publications we reviewed made sense of ‘evidence gaps’ was by emphasising the problem of too many, or imprecise, definitions for fakes. As critical commentators Cloatre (2016) and Quet (2018) note, the concept of fake or counterfeit medicines is strangely ill-defined, even in formal settings. In the case of fake or counterfeit medicines, blurry legal definitions construct corollary ontological instabilities. This is despite several decades’ worth of attempts by organisations such as the World Health Organisation to produce meaningful categories, as Christopher Sirrs’ meticulous archival research shows (2019). In other words, the problem of a lack of clear definitions, alongside a proliferation of recognised categories of non-normative drug compounds, is something all scholars face when generating and collating data on which to base their claims. How scholars manage trouble with categories when researching fakes affects which world view and understanding of the issue is made visible (Bowker & Star, 1999).

To remedy the problem of too many, or ineffective, definitions, which makes evidential claims about fakes difficult, many global public health articles offer new terminologies. Harris and colleagues (2009) write of this trouble and their decision to use the catch-all of ‘fakes’:

Counterfeit and substandard drugs are a serious and growing problem around the world – especially in less developed countries. There are many reasons for this, including imitation, inappropriate packaging, poor manufacturing processes, and improper conditions during transportation and storage. At the point of purchase, such drugs share the common feature that they are not what they purport to be, so for simplicity we class them all as ‘fakes’ in this paper. (p. 4)

Definitional troubles are a long-standing issue in the study of fakes. This is hardly an isolated instance of struggling with the lived distance between distinct regulatory definitions (e.g. substandard, unlicensed, counterfeit) and consumers’ understandings of the safety or efficacy of different kinds of non-normative drugs, but rather indicates the ontological trouble of categorising fakes. For instance, different places use different categories, and definitions are affected by highlighted specific and situated circumstances:

International efforts to combat fake medicines have been hampered by the inability of countries to agree in a legal definition of fake medicines. Legal scholars and health experts are now pushing for a global treaty to correct this problem and allow for greater cooperation between national authorities, but this will be many years off. (Mydin and Stevens, 2013, p. 1)

Categories are also shaped by discipline and field of use. Experts in pharmacology or global public health also encounter difficulties when using legal and regulatory categories in accordance with their technical definitions. Another term used in the published literature is ‘counterfeit’ – itself an intellectual property term – which refers to purposefully criminal activity and implies that drugs are unsafe. For example, in describing an incident in India in which a vial of eye medication revealed microbial contamination and no active ingredient a paper refers to the medication as ‘counterfeit’ rather than ‘spurious’ (as per the Indian Legal Code) or ‘falsified’ (as per the WHO's term) (Stewart et al., 2016). Whilst the meaning of counterfeit may be popularly understood as both ‘criminal’ and ‘unsafe’, this is technically incorrect. To manufacture a counterfeit drug is certainly an illegal act, but it is generally a civil offence: intellectual property theft. It is common knowledge among those familiar with pharmaceutical trade and policy a drug that is manufactured and distributed without having first purchased (owning) the rights to do so could be identical to the ‘real’ thing in terms of its therapeutic efficacy. The typical case of this is that of a generic drug.

Although legal definitions exist, how to make these function in practice is an ongoing challenge and has led to the introduction of new terms and the removal of others (Sirrs, 2019). Disagreement over definitions and the need to seek clarity regarding what kinds of fakes are of greater concern usually result in requests for more ‘real data’ through improved surveillance, monitoring, and global alert systems, something recently recognised by the WHO (2017b), which continues to seek to simplify terminology. Nevertheless, for the purposes of this article, our point is that efforts to craft better terminology still leaves under-investigated the basic question of the evidentiary basis of claims about fake drugs. The problem of evidence cannot be addressed by better terminology alone (see Herrick, 2017).

## Discussion: Constructing an unknowable problem?

Our research into the way claims about fake drugs are made and mobilised could, we suspect, be applied to other public health issues with a similar result. However, what this paper emphasises is the cumulative effect of a series of rhetorical practices in global health research of fakes through the vehicle of the published article and other recognised forms of high-quality research (Latour, 1987). By locating the problem of fakes, as well as its possible solution, in data and evidence, together the articles we review signal the possibility of knowing fakes. Yet, our analysis shows that, paradoxically, the ways in which fakes are currently being framed and talked about in science simultaneously results in their unknowability.

Let us be clear: to call fake drugs an ‘unknowable problem’ is not to discount or dismiss the possibility that consuming inefficacious drugs may cause substantial harm to health. But our review of the literature tells us that – while papers regularly voice concern about the urgent problems that fake drugs present – there is no consensus on what counts as an *‘evidence base’*, neither in terms of the scope nor the effects of fake drugs on global public health. In particular, we were struck by the implicit assumption conveyed by much of the scholarship that there is no utility in questioning the evidence gaps themselves.

For instance, published papers and policy reports always declared the importance of the problem of fakes whilst acknowledging the lack of systematically collected data with sufficient sample sizes and random sampling design (e.g. WHO, 2017). Our review showed that scientific publications on fakes consistently chose not to question the *conditions* that produced such a paucity of data. The same WHO (2017) report, for instance, proposes developing superior technical methods rather than approaching research about fakes differently, despite other work in the field that has pointed to the pharmaceutical industry severely constraining the circulation of data (e.g. Sismondo, 2018). Most pharmaceutical data is produced, collected, and held by manufacturers – and classified as ‘proprietary’ – yet this lack of access to data finds no place in the vast majority of global public health scholarship about the problem of fake drugs (Sismondo, 2018). Data on drug quality is often held by manufacturers and is unavailable for independent review by researchers. The availability of data about drug quality is also hampered by the fact that, until very recently, independent testing of drugs is typically prohibitively expensive for independent (that is, non-industry) researchers. In other words, it is not just that people *don't* use data, nor simply that data is absent. Rather, it appears that data is, to a large degree, inaccessible to scholars.

Taken together, our findings point to an *oversupply* of curiosity about how fake drugs exist without leaving clear evidence alongside a corollary *undersupply* of curiosity about how and why evidence gaps condition the possibility for knowledge about fake drugs. Our analysis shows that this regular reiteration of urgency in the absence of good data has led to limited innovation in research questions or designs.^2^ Instead, the cumulative effect is that scientific research is constructing the ‘problem of fake drugs’ as *knowable* in a way that forecloses a discussion of the politics and circulation of data and evidence. We argue that rather than being ‘unknown’, and therefore a problem that can be known (and resolved) through more research, taken together, one of the most powerful effects of this body of scholarship has been the elision of other approaches and ways of thinking about fakes.^3^

## Conclusion: The ghost in the data

In closing, we would like to return to the title of this article. What is ‘ghost data’? By invoking the term ‘ghost data’, we are conscious that we are using it slightly differently compared to how it functions in other disciplines. Statisticians use the concept of ‘ghost data’ to refer to data that are not actually there. As John Sall (quotes in Mejdal, 2017) explains, ‘Just as we appreciate the data we have, we also need an appreciation of data that isn't there. We need to know how to handle it, know how to model with it, and put it to work’. Statisticians and others depend on the idea of ghost data in order to model a fuller picture of non-existent and under-reported data, for example in mortgage data:

Mortgage data for the years before the financial crisis of 2008 is a good example of informative missing [data]. When modelling the probability of a mortgage default, the results are biased if all the rows that have missing values for variables like “income” and “debt-to-income ratio” have been dropped. Often the loan applicant knew that providing those values would result in failure to qualify for the loan, so the values were omitted. In the data table we supply with our sample data, the missingness of debt-to-income ratio is the most important predictor of mortgage default. (Mejdal, 2017, p. X)

In other words, missing data itself can inform the picture, creating patterns that can produce new knowledge. This is not a secret. In this sense, ghost data is very similar to the work that proxies do in much epidemiological research. Indeed, mastering the art of quantitative analysis necessarily demands that researchers acknowledge and determine the role played by ghost data.

In scholarly publishing on fake drugs, however, the role of ghost data is different. In much of the scholarship we analysed, ghost data itself functions as justification for research. In other words, in the production of knowledge about fake drugs within global public health, ghost data is a different kind of problem and refers to the myth-like status of fake drugs within global public health. We wish to reiterate that we do not claim that fake drugs do not exist, nor do we claim that the circulation of all kinds of substandard or low-quality drugs does not exist. And especially, we do not claim that poor-quality drugs cannot have a negative effect on health outcomes. But while there is broad agreement that fake drugs may present a problem, there is little, if any, consensus regarding what kind of problem and for whom. Fake drugs are, at the very least, an awkward object of study: the scientific publications we reviewed imply that there are clear and knowable boundaries between ‘real’ and ‘fake’, yet simultaneously demonstrate a conviction that the trade in fake drugs is nearly impossible to spot, quantify, or prevent. Still, research continues to make new data and claims about fakes as a significant global health problem.

In this paper, we have conducted an epistemic audit of what is gained and what is lost in the current state of play of global public health knowledge about fake drugs. We have considered what the larger epistemic effects of these persistent evidence gaps might be and concluded that these evidence gaps *actually function* as a ghost in the data that might be misdirecting attention (Peeters Grietens et al., 2019). This matters because by generating more data and highlighting absences we are hindered from understanding the power dynamics that condition how we know fakes, such as how quality assurance data (or, indeed its absence) structures the contemporary political economy of pharmaceuticals within global health.

Were we writing a different paper – one which charted the rise in attention to worries about fake drugs alongside the politics of the global trade in pharmaceuticals, we surely would have discussed the ways in which attempts to regulate this trade have played out over the course of the last few decades. In particular, we would have paid attention to the 1995 agreement on Trade-Related Aspects of Intellectual Property Rights (TRIPS), as well as the respective careers of the World Trade Organization (WTO) and the United States’ Food and Drug Administration (USFDA). Such an account would also have considered the various legal battles that were waged over the regulation of international trade in pharmaceuticals in the face of the growth of India's export commerce in low-cost generic exports during this period, particularly in the wake of the AIDS crisis in Africa. And, were we writing that other article we would certainly have attempted to account for the rise of fake-talk in global public health scholarship on pharmaceuticals as part and part of this other history of the international political economy of pharmaceuticals – that is, how the globalisation of the pharmaceutical trade has regularly served as an international flashpoint, illuminating tensions between states and markets.

As we signalled earlier in this article, civil society organisations such as Third World Network (TWN) have been particularly active and we would refer readers to their website to access their publications: https://www.twn.my/. The work of TWN suggest the assumption that fake drugs constitute an urgent and life threatening danger feeds into the past several decades’ attempts to regulate global trade in pharmaceuticals. Similarly, wide-spread suspicions about the safety of the global pharmaceutical supply has been shown to serve the interests of some countries’ pharmaceutical sectors, whilst undermining the interests of others.

Whilst these debates are significant, our task in this paper has been to investigate how fake drugs have become a widely accepted concern in global public health research. Along the way, many colleagues asked if our research served to ‘expose big pharma’ – that is, to document collusion between scholars and industry. Although other academics have demonstrated this meticulously (e.g. Sismondo, 2018), this was neither our question not our aim. We neither looked for nor found a nexus between scholars and pharmaceutical manufacturers. Even though this dynamic exists in the world of scholarly publishing (as Sismondo convincingly demonstrates), it does not seem plausible that it could account for all of the publications we came across in which claims about the existence and effects of fake drugs in absence of robust evidence.

Instead, our epistemic audit of global public health scholarship on fake drugs suggests that this scholarship is both shaped and sustained by cultures of knowledge production. As with all forms of culture, these cultures of knowledge production have some assumptions at their core. The key assumptions of this culture of scholarship is that the oft-repeated claims about the urgent dangers presented by fake drugs is self-evident. We suggest that one significant effect of these assumptions is that research does not focus on why fakes are difficult to measure, evidence and conceptualise and for these assumptions to be worthy of interrogation. In particular, we note that global health scholarship is not, in the main, asking questions about the conditions that foreclose access to data. Nor are the papers published questioning their assumptions and about the self-evident nature of ‘the problem of fake drugs’. We are concerned that not questioning these assumptions in the face of what we refer to as ‘evidence gaps’ may in and of itself mitigate against the very possibility of realising any ‘truth’ of fake drugs. By bringing sociological, anthropological and historical methodologies to bear on understanding this culture of knowledge, we suggest that global health research may have much to gain by fostering a more critical gaze towards the structural forces and material conditions that shape what we know and how we come to know it.

## Data Availability

See ‘Appendix’

## Notes

1 The on-going research project, ‘What's at stake in the fake? Indian pharmaceuticals, African markets and global health’, charts the rise of worries about fake drugs – particularly worries about the safety of Indian pharmaceutical exports as they circulate in African markets – and assesses the impact that these worries have had across policy and research communities, as well as among consumers: ‘What's at stake in the fake? Indian pharmaceuticals, African markets and global health’. Wellcome Trust Collaborative Award in the Humanities and Social Sciences 212584/Z/18/Z. Investigators: Sarah Hodges, Julia Hornberger, Rene Gerrets.

2 A notable exception is Pisani et al. (2019). Their work, grounded in political economy (that is, the relationship between states and markets), points to how it might be possible to target real-time in market quality sampling in ways that look promising.

3 It is precisely this finding that forms the basis for the research questions at the heart of the on-going research project, ‘What's at stake in the fake? Indian pharmaceuticals, African markets and global health’ (2018–2023).

## Appendix of sources consulted in review

‘Counterfeit drugs: A growing global threat.’ *Lancet* [editorial] (25 February–2 March 2012) 379, 9817: 685.

Almuzaini, T., Choonara, I., & Sammons, H. (2013). Substandard and counterfeit medicines: A systematic review of the literature. *BMJ Open* 3: e002923.

Banerjee, Y. (2015). Mission Indradhanush and the counterfeit drug trade in India. *Lancet*, 15(12): 1379–1380.

Bate, R., Jensen, P., Hess, K., Mooney, L., Milligan, J. (2013). Substandard and falsified anti-tuberculosis drugs: a preliminary field analysis. *International Journal of Tuberculosis and Lung Disease* 17(3): 308–311.

Clark, F. (2015). Rise in online pharmacies sees counterfeit drugs go global. *Lancet,* 386 (10001): 1327–1328.

Fatokun, O. (2016). Curbing the circulation of counterfeit medicines in Nigeria. *Lancet*, 388 (10060): 2603.

Glass, B. (2014). Counterfeit drugs and medical devices in developing countries. *Research and Reports in Tropical Medicine*, 5: 11–22.

Gwatidzo, S.D., Murambinda, P.K., & Makoni, Z. (2017). Medicines counterfeiting in Africa: A view from Zimbabwe. *Med Access @ Point Care*, 1, 1: e82-e86.

Harris, J., Stevens, P., & Morris, J. (2009). Keeping it real: Combating the spread of fake drugs in poor countries. Retrieved from: http://www.ncpa.org/sub/dpd/index.php?Article_ID=18062

Koczwara, A., & Dressman, J. (2017). Poor-quality and counterfeit drugs: A systematic assessment of prevalence and risks based on data published from 2007 to 2016. *Journal of Pharmaceutical Sciences* 106 (10): 2921–2929.

Kaur, H., Clarke, S., Lalani, M., Phanouvong, S., Guerin, P., McLoughlin, A., Wilson, B.K., Deats, M., Plancon, A., Hopkins, H., Mianda, D., Schellenberg, D. (2016). Fake anti- malarials: Start with the facts. *Malaria Journal* 15: 86.

Kelesidis, T. & Falagas, M. E. (2015). Substandard/counterfeit antimicrobial drugs. *Clinical Microbioly Review,* 28(2): 443–464.

Khan A.N, & Khar, R.K. (2015). Current scenario of spurious and substandard medicines in India: A systematic review. *Indian Journal of Pharmaceutical Sciences.* 77(1): 2–11.

Interpol Pharmaceutical Crime Directorate. (2014). 17th July 2014. Pharmaceutical crime and organized criminal groups: an analysis of the involvement of organized criminal groups in pharmaceutical crime since 2008. Interpol HQ, Lyon, France.

Mackey, T.K. & Liang, B.A. (2011). The global counterfeit drug trade: patient safety and public health risks. *Journal of Pharmaceutical Sciences,* 100: 4571–4579.

Mackey, T.K., & Liang. B.A. (2013). Improving global health governance to combat counterfeit medicines: a proposal for a UNODC-WHO-Interpol trilateral mechanism. BMC Med 11: 233.

Mackey, T.K., Liang, B.A., York, P., Kubic, T. (2015). Counterfeit drug penetration into global legitimate medicine supply chains: A global assessment. *American Journal of Tropical Medicine and Hygiene*, 92 (6 Supple): 59–67.

Medina, E., Bel, E., Suñé, J.M. (2016). Counterfeit medicines in Peru: A retrospective review (1997–2014). *BMJ Open,* 6 (4): e010387.

Nayyar, G.M., Breman, J.G., newton, P.N., Herrington, J. (2012). Poor quality antimalarial drugs in south-east Asia and sub-Saharan Africa. *Lancet,* 12 (6): 488–496.

Nayyar, G.M.L., Attaran, A., Clark, J.P., Culzoni, M.J., Fernandez, F.M., Herrington, J.E., Kendall, M., Newton, P.N., & Breman, J.G. (2015). Responding to the pandemic of falsified medicines. *American Journal of Tropical Medicine* Hygiene, 92 (6 Suppl):113–118.

Newton, P., Hanson, K., & Goodman, C. (2017). Do anti-malarials in Africa meet quality standards? The market penetration of non-quality assured artemisinin combination therapy in eight African countries*. Malaria Journal,* 16 (204): 1–31.

Ossola, A. (2015). The fake drug industry is exploding, and we can't do anything about it. *Newsweek*. 9/17/15 at 6:55 AM

Pisani, E. (2015). Antimicrobial resistance: What does medical quality have to do with it? *Review of Antimicrobial Resistance*.

Pisani, E. (2016). Why are we losing the war on bugs. *Prospect Magazine* 239. Retrieved from: https://www.prospectmagazine.co.uk/magazine/losing-the-war-on-bugs-low-quality-medicines-tropical-illnesses

Pisani, E., Nistor, A-L., Hasnida, A., Parmaksiz, K., Xu, J., & Kok, M.O. (2019). Identifying market risk for substandard and falsified medicines: an analytic framework based on qualitative research in China, Indonesia, Turkey and Romania. https://wellcomeopenresearch.org/articles/4-70/v1

Rahman, M.S., Yoshida, N., Tsuboi, H., Tomizu, N., Endo J., Miyu, O., Akimoto, Y., Kimura, K. (2018). The health consequences of falsified medicines: a study of the published literature. *Tropical Medicine International Health*, 23(12): 1294–1303.

Shukla, N., & Sangal, T. (2009). Generic Drug Industry in India: The Counterfeit Spin. *Journal of Intellectual Property* Rights 14: 236–240.

Stevens, P. Haja Mydin, H. (2013). Fake medicines in Asia. *EMHN Briefing No. 1:* 1–8.

Stewart, M. W., Narayanan, R., Gupta, V., Rosenfeld, P.J., Martin, D.F. Chakravarthy, U. (2016). Counterfeit Avastin in India: Punish the criminals, not the patients. *American Journal of Opthalmology*, 170: 228–231.

Verma, S., Kumar, K., & Philip, P.J. (2014). The Business of Counterfeit Drugs in India: A Critical Evaluation. *International Journal of Management and International Business Studies*, 4 (2): 141–148.

Wertheimer, A., Norris, J. (2009). Safeguarding against substandard/ counterfeit drugs: mitigating a macroeconomic pandemic. *Res Social Adm Pharm* 5: 4–16.

World Health Organization (2017a). A study on the public health and socioeconomic impact of substandard and falsified medicines. WHO reference number: WHO/EMP/RHT/2017.02

World Health Organization (2017b). *WHO Global Surveillance and Monitoring System for substandard and falsified medical products*. Geneva: World Health Organization. WHO reference number: WHO/EMP/RHT/2017.01
